# Diagnosis of Forme Fruste Keratoconus Using Corvis ST Sequences with Digital Image Correlation and Machine Learning

**DOI:** 10.3390/bioengineering11050429

**Published:** 2024-04-26

**Authors:** Lanting Yang, Kehan Qi, Peipei Zhang, Jiaxuan Cheng, Hera Soha, Yun Jin, Haochen Ci, Xianling Zheng, Bo Wang, Yue Mei, Shihao Chen, Junjie Wang

**Affiliations:** 1 National Engineering Research Center of Ophthalmology and Optometry, Eye Hospital, Wenzhou Medical University, Wenzhou 325027, China; 2 State Key Laboratory of Ophthalmology, Optometry and Visual Science, Eye Hospital, Wenzhou Medical University, Wenzhou 325027, China; 3 National Clinical Research Center for Ocular Diseases, Eye Hospital, Wenzhou Medical University, Wenzhou 325027, China; 4 Shenzhen Institute of Advanced Technology, Chinese Academy of Sciences, Shenzhen 518055, China; 5 University of Chinese Academy of Sciences, Beijing 101408, China; 6 State Key Laboratory of Structural Analysis, Optimization and CAE Software for Industrial Equipment, Department of Engineering Mechanics, Dalian University of Technology, Dalian 116023, China; 7 International Research Center for Computational Mechanics, Dalian University of Technology, Dalian 116023, China; 8 Department of Ophthalmology, Sichuan Mental Health Center, Mianyang 621054, China

**Keywords:** forme fruste keratoconus, digital image correlation, machine learning, corvis ST

## Abstract

Purpose: This study aimed to employ the incremental digital image correlation (DIC) method to obtain displacement and strain field data of the cornea from Corvis ST (CVS) sequences and access the performance of embedding these biomechanical data with machine learning models to distinguish forme fruste keratoconus (FFKC) from normal corneas. Methods: 100 subjects were categorized into normal (N = 50) and FFKC (N = 50) groups. Image sequences depicting the horizontal cross-section of the human cornea under air puff were captured using the Corvis ST tonometer. The high-speed evolution of full-field corneal displacement, strain, velocity, and strain rate was reconstructed utilizing the incremental DIC approach. Maximum (max-) and average (ave-) values of full-field displacement V, shear strain γxy, velocity VR, and shear strain rate γxyR were determined over time, generating eight evolution curves denoting max-V, max-γxy, max-VR, max-γxyR, ave-V, ave-γxy, ave-VR, and ave-γxyR, respectively. These evolution data were inputted into two machine learning (ML) models, specifically Naïve Bayes (NB) and Random Forest (RF) models, which were subsequently employed to construct a voting classifier. The performance of the models in diagnosing FFKC from normal corneas was compared to existing CVS parameters. Results: The Normal group and the FFKC group each included 50 eyes. The FFKC group did not differ from healthy controls for age (*p* = 0.26) and gender (*p* = 0.36) at baseline, but they had significantly lower bIOP (*p* < 0.001) and thinner central cornea thickness (CCT) (*p* < 0.001). The results demonstrated that the proposed voting ensemble model yielded the highest performance with an AUC of 1.00, followed by the RF model with an AUC of 0.99. Radius and A2 Time emerged as the best-performing CVS parameters with AUC values of 0.948 and 0.938, respectively. Nonetheless, no existing Corvis ST parameters outperformed the ML models. A progressive enhancement in performance of the ML models was observed with incremental time points during the corneal deformation. Conclusion: This study represents the first instance where displacement and strain data following incremental DIC analysis of Corvis ST images were integrated with machine learning models to effectively differentiate FFKC corneas from normal ones, achieving superior accuracy compared to existing CVS parameters. Considering biomechanical responses of the inner cornea and their temporal pattern changes may significantly improve the early detection of keratoconus.

## 1. Introduction

Keratoconus (KC) is a corneal disease characterized as gradual, non-inflammatory thinning and conical protrusion of the cornea. The prevalence and incidence rates of KC have been estimated to range from 0.2 to 4790 per 100,000 people and 1.5 to 25 per 100,000 people/year, respectively, and Asians have a much higher frequency and incidence than Caucasians [1]. KC usually manifests in adolescence or early adulthood as myopia and irregular astigmatism which may significantly reduce visual quality [2] and require corneal transplantation in severe cases [3]. Although one eye may be affected initially, KC is a progressive condition that eventually affects both eyes [4,5].

The early detection of KC is vital to slow or stop its progression typically by corneal cross-linking procedures. However, this has proven to be challenging because the disease, at its earliest stage of forme fruste keratoconus (FFKC), may present normal morphologic features through tomography examinations [1]. Such a challenge has increasing clinical significance at the current heat of laser vision corrections (LVCs), such as TPRK, LASIK and SMILE, where undetected FFKC has become one of the most important risk factors of keratectasia following LVCs [6]. Identifying FFKC allows for the early intervention and management of KC patients. First, early detection can lead to timely treatments, such as corneal collagen cross-linking or specialized contact lenses, which can potentially halt or slow the progression of the disease to more advanced stages. Second, timely diagnosis and management can prevent significant visual impairment associated with progressive keratoconus. By addressing the condition early on, vision loss and the need for more invasive treatments, like corneal transplantation, can be minimized. Third, a precise diagnosis provides patients with a clearer understanding of their condition, its implications, and the importance of regular follow-ups. This facilitates better patient education and counseling, empowering patients to make informed decisions about their eye health. Most importantly, accurate diagnosis of FFKC enables ophthalmologists to tailor treatment plans according to the individual’s specific condition and progression rate. This personalized approach can optimize treatment outcomes and patient satisfaction [7].

Conventionally, the diagnosis of KC (including FFKC) was based on corneal topography or tomography, and significant efforts have been made to develop novel indices based on morphologic features to reveal the earliest signs of disease development [8]. With the development of machine learning in medical data application [9,10,11], it is now of great interest to employ AI algorithms in FFKC diagnosis. Notably, recent developments in artificial intelligence (AI) techniques have enabled new attempts to combine multiple morphologic features for such purposes, for example, Hidalgo et al. used SVM (Support Vector Machine), RF (Random Forest), and other artificial intelligence models to differentiate FFKC from normal and KC corneas, with some achieving an AUC higher than 0.9 but the sensitivity remained below 90% [12,13].

In recent years, corneal biomechanics has shined new light on FFKC diagnosis. On one hand, experimental studies proved that local biomechanical weakening of the cornea precedes its morphologic irregularities [1]; on the other, the Corneal Response Analyzer (ORA) and Corvis ST (CVS) have enabled in vivo corneal biomechanical evaluations (in addition to the conventional tomography) and multiple studies have demonstrated the added value of such evaluations in improving the accurate detection of KC [14]. With the enriched diagnostic information, Yan Wang et al. trained an AI model to distinguish KC from normal corneas and achieved an accuracy of 98.7%, a sensitivity of 97.4%, a specificity of 100%, and a precision of 100% in the external validation set [15]. Nan-Ji Lu et al. demonstrated that combining corneal morphologic and biomechanical variables could increase the diagnostic effectiveness of AI models [16].

By recording the deformation of the cornea during the air puff test, the CVS can provide more biomechanical measures of the cornea than the ORA, which have been the foundations for the recent development of various indices intended to reflect early and subtle biomechanical abnormalities of the cornea [17]. However, most CVS parameters are based on the deformation information of the anterior corneal surface and only focus on one or two time points during the entire course of the deformation [18,19,20]. First, the subtle local irregularities inside the cornea may not be reflected due to the superficial nature of existing parameters. Second, as a visco-elastic material, the deformation of the cornea is highly time-dependent [21,22,23,24], meaning the deformation patterns of the cornea during the entire test (inward deformation and outward restoration) can be a significant indicator of subtle abnormality. Thus, tracking the entire bulk of the cornea and its whole deformation history may provide more sensitive information that reflects the local and subtle biomechanical weakening which may be otherwise overlooked by surface-based biomechanical indices and tomographic indices.

In this study, an incremental digital image correlation method (previously developed by the same group of the current study [25]) was used to obtain the deformation and strain patterns of the entire corneal cross-section during the whole air puff test. These forms of information consisting of both spatial and temporal domains were then inputted to several AI models to create a framework that distinguished between FFKC and normal corneas. The performance of these AI models was then compared with existing CVS parameters.

## 2. Material and Methods

### 2.1. Participants and Procedures

The study collected preoperative Corvis ST test results of 50 healthy participants for the normal group and 50 FFKC patients for the FFKC group. The test results of the normal group were collected from patients presenting for laser vision correction in the Eye Hospital of Wenzhou Medical University, using the inclusion criteria of no ocular or systemic abnormalities, no ocular surgery history, a stable-corrected-distance visual acuity (CDVA) of 20/20 or better for 2 years before surgery.

For the FFKC group, the inclusion criteria were (1) the contralateral eye was diagnosed as KC (at least one slit-lamp finding (Fleischer ring, Vogt striae, or central thinning) and two signs of keratoconus using Pentacam tomography (Oculus Optikgeräte GmbH, Wetzlar, Germany), such as decreased thinnest pachymetry, skewed asymmetric bowtie/inferior steep or increased inferior steepness), (2) CDVA of 20/20 or better, (3) no keratoconus signs on slit-lamp examination, (4) maximum keratometry (Kmax) less than 47.40 diopters (D), (5) thinnest pachymetry of 480 µm or greater obtained by Pentacam, and (6) “normal” topography with the difference between the Kmax values in the inferior and superior areas at 3 mm (I-S value) less than 1.40 D, no skewed asymmetric bowtie/inferior steep, and keratoconus percentage index (KISA%) less than 60.

Visual acuity, objective, and manifest refraction, as well as demographic information including participant age and gender, were recorded. Fundoscopy, noncontact intraocular pressure, and slit-lamp microscopy were also carried out. Corneal biomechanics were assessed using the Corvis ST (Oculus Optikgeräte GmbH, Wetzlar, Germany) and the pachymetry using Pentacam; only quality tests with “OK” were kept for analysis.

The study was approved by the Research Ethics Committee of Eye Hospital of Wenzhou Medical University (2022-198-K-154) and conducted following the tenets of the Declaration of Helsinki.

### 2.2. Incremental DIC Method

**Figure 1:**
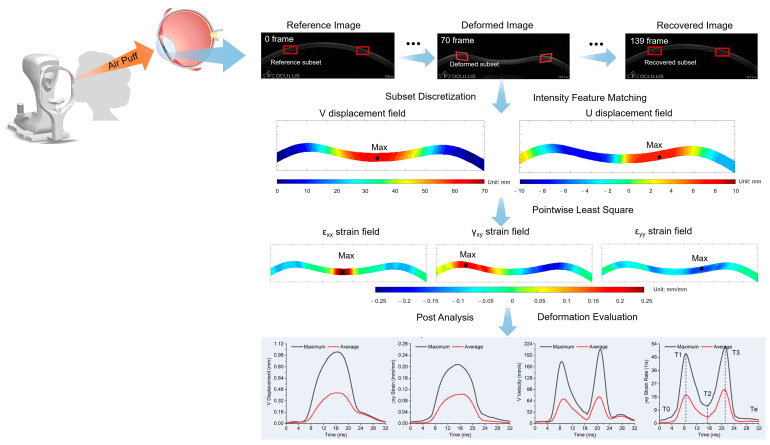
Schematic graph of the incremental digital image correlation process and the resulting biomechanical measures.

To assess full-field displacement, strain, velocity, and strain rate, the 140 images from the whole deformation series were loaded into the incremental DIC program [26], where full-field displacements in the horizontal (U) and vertical (V) directions were tracked, which were used to estimate the three Cauchy strain components (ε_xx_, ε_yy_, and γ_xy_) using the pointwise least-square method. The finite differential approach was then used to determine two velocity components (UR and VR) and three strain rate components (ε_xx_R, ε_yy_R, and γ_xy_R). The details of the analysis can be found in the previous study published by the same group [25]. The entire process can be seen in Figure 1.

### 2.3. Feature Extraction and Model Construction

The maximum (max-) and average (ave-) values of the full-field displacement V, shear strain γ_xy_, velocity VR, and shear strain rate γ_xy_R were retrieved over time (whole 140 frames, lasting approximately 30 ms [25]), resulting in 8 evolution curves against time (Figure 1, bottom row), denoted as max-V, max-γ_xy_, max-VR, max-γ_xy_R, ave-V, ave-γ_xy_, ave-VR, and ave-γ_xy_R, respectively.

These evolution data were inputted to two machine learning (ML) models, namely, Naïve Bayes (NB) [27] and Random Forest (RF) [28]. A voting classifier was then constructed with these models to balance out their individual weaknesses and ensure a relatively stable result. Meanwhile, a logistic regression model was also trained. The whole dataset of 100 patients was divided into a training set (80%) for model training and a validation set (20%) for reliability assessment.

### 2.4. Experimental Setting and Performance Metrics

All calculations were conducted using a Windows 10 operating system equipped with 8 GB RAM and an Intel Core i5 processor running at 1.6 GHz and featuring 8 CPUs. The built-in functions inside the sklearn package were used in the Python 3.7 environment.

The performance metrics used to evaluate the efficacy of the machine learning models were accuracy, precision, recall, and F1 score. All these metrics were based on the values of the confusion matrix.

(1) $$Accuracy=\frac{TP+TN}{TP+TN+FP+FN}$$

(2) $$Precision=\frac{TP}{TP+FP}$$

(3) $$Recall=\frac{TP}{TP+FN}$$

(4) $$F1\;score=2\times \frac{Precision\times Recall}{Precison+Recall}$$

Receiver operating characteristic (ROC) analysis was also carried out which enabled comparisons in diagnostic effectiveness between the ML models and existing CVS parameters.

### 2.5. Statistical Analysis

Statistical analysis was conducted using SPSS software (version 24; IBM Corporation, Armonk, NY, USA: IBM Corp). Descriptive statistics were reported as mean ± standard deviation. The demographic data of both groups were compared for differences. Two independent sample *t*-tests were used for continuous variables, whereas Chi-square tests were adopted for categorical data. A significance level of *p* < 0.05 was considered statistically significant for all tests. For the CVS parameters, ROC curves and area under the curve (AUC) were utilized to determine their optimal cut-off values, sensitivity, and specificity in distinguishing FFKC from the normal corneas.

## 3. Results

### 3.1. The Demographic Data of the Participants

The demographic data of the participants are listed in Table 1. The FFKC group did not differ from healthy controls in terms of age (*p* = 0.26) and gender (*p* = 0.36) at baseline, but they had a significantly lower bIOP (*p* < 0.001) and a thinner central cornea thickness (CCT) (*p* < 0.001).

### 3.2. Initial Model Construction

The eight time-dependent evolution curves from the incremental DIC method, namely, the maximal and average values of the vertical displacement V, shear strain γ_xy_, vertical velocity VR, and shear strain rate γ_xy_R, were inputted to the two ML models (NB and RF). The initial results indicated that including both maximal and average values did not lead to accurately distinguishing between FFKC and normal groups. After a few attempts to cull and add data forms, only the average values were kept, which led to a significantly better diagnosis performance, probably due to the higher calculation noise levels of the maximal values. A voting classifier model (NB + RF) was subsequently employed to differentiate FFKC from normal corneas. During these initial attempts, datasets with different numbers of time points (from 1 time point to the full 140 points) were incrementally incorporated into the models and a gradual increase in performance was observed with an increasing number of time points (Supplementary Figures S1 and S2); the results with the full dataset (140 time points) are reported herein and were compared to existing CVS parameters. The feature importance of the ML models is shown in Supplementary Figure S3.

### 3.3. Performance of the Machine Learning Models and Voting Classifier Model

The accuracy, precision, recall score, F1-score sensitivity, specificity, and AUC values of the machine learning (both the training and validation datasets) are shown in Table 2. The results demonstrate that the proposed voting ensemble model had the best performance with an AUC of 1.00, followed by the RF model with an AUC of 0.99. The 5-fold cross-validation results of the ML models are shown in Supplementary Table S1, with the AUCs of the voting and RF models both reaching 0.90.

### 3.4. Performance Comparison with Existing CVS Parameters

**Figure 2:**
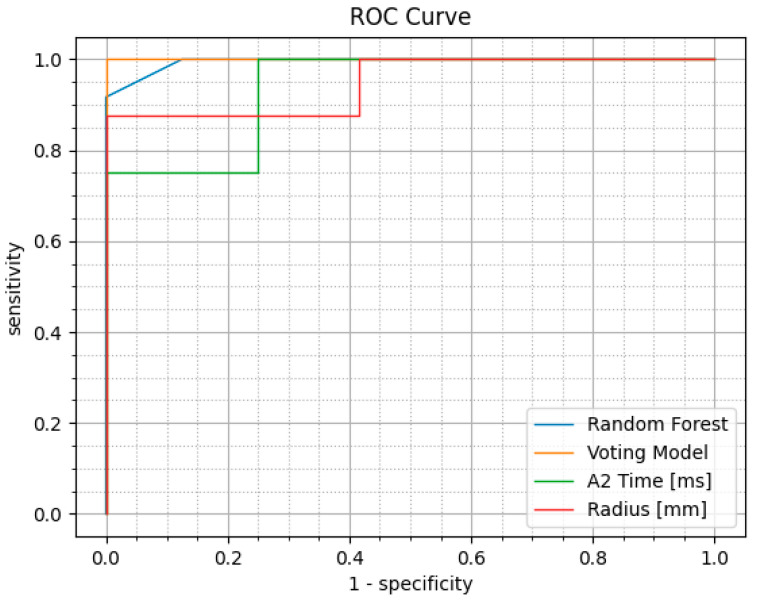
The ROC curves of the top two Corvis ST parameters and the top two ML models.

The ROC analysis results of the top 10 existing CVS parameters with the validation dataset are presented in Table 3. Radius and A2 Time were among the best performing parameters with an AUC of 0.948 and 0.938, respectively. However, no existing parameters performed better than the AI models, where the voting classifier and RF had an AUC of 1.00 and 0.99, respectively (Table 2). The ROC curve of the NB, voting classifier, Radius, and A2 Time are shown in Figure 2. The complete ROC results of existing CVS parameters are presented in Supplementary Table S2.

## 4. Discussion

In this study, displacement and strain data across the entire corneal cross-section were calculated from image sequences capturing corneal deformation throughout the air puff test, employing the incremental DIC method. These datasets, comprising spatial and temporal biomechanical information of the cornea, were utilized as inputs for various machine learning models to discriminate between forme fruste keratoconus (FFKC) and normal corneas, yielding promising outcomes compared to existing CVS biomechanical parameters. Both time and spatial domain data, including inner corneal biomechanical properties, were integrated into the FFKC diagnosis from a pure biomechanical perspective without recourse to topography. In the context of the definition of FFKC, as delineated in most related research, FFKC is characterized by its proximity to the normal cornea and represents an early stage in the development of keratoconus compared to subclinical KC [12,13,16,29]. As the current study focused on diagnosing FFKC from normal corneas, the discussion herein thus excluded studies that treated subclinical KC and FFKC as equivalent corneal conditions.

The timely and precise diagnosis of FFKC has garnered recent research attention, driven by its escalating clinical significance, and facilitated by advancements in tomographic and in vivo biomechanical measurement techniques, and, notably, the expanding utilization of AI models for ocular medical imaging data analysis.

Smadja et al. utilized a decision tree model to distinguish FFKC from normal and keratoconus corneas, achieving a sensitivity of 93.6% and a specificity of 97.2% based on morphological features obtained from the GALILEI system [29]. Hidalgo et al. employed an SVM model to differentiate FFKC from normal and keratoconus corneas, attaining an AUC of 0.922, with a sensitivity and a specificity of 79.1% and 97.9%, respectively, based on morphological features obtained from Pentacam [12]. Kovács et al. constructed a neural network model using morphological data derived from a Scheimpflug camera, achieving an AUC of 0.97, a sensitivity of 90%, and a specificity of 90% in distinguishing FFKC from normal and keratoconus corneas [13]. Rachana et al. utilized Bowman’s topography data derived from OCT and employed a Random Forest model to differentiate FFKC from normal and keratoconus corneas, yielding an AUC of 0.95, a sensitivity of 72.2%, and a specificity of 95.64% [30]. However, none of these studies exclusively differentiated FFKC from normal corneas only, but rather from a mixed population of both normal corneas and KC, and the uneven sample sizes among the groups may have impacted the diagnostic performance of the models. In clinical practice, the accurate differentiation between FFKC and normal corneas is of primary concern.

Recently, a study employed a Random Forest model to integrate both the biomechanical information (CVS) and morphological features (SD-optical coherence tomography) of the cornea to distinguish FFKC from normal corneas, achieving an AUC of 0.902, with a sensitivity and a specificity of 73.53% and 87.74%, respectively [31]. This study, being different from those mentioned earlier, leveraged the corneal biomechanical properties for the early detection of KC, which aligns with the understanding of keratoconus pathogenesis that a corneal biomechanical abnormality may precede structural alterations detectable through traditional slit-lamp examination and corneal topography [32]. Notably, the biomechanical characteristics of the cornea undergo changes during the early stages of keratoconus, with corneal stiffness progressively diminishing as the condition advances [33,34]. As such, recent years have seen numerous investigations using corneal biomechanics to aid KC detections.

The Ocular Response Analyzer (ORA) is the first clinical device that enables in vivo corneal biomechanical assessment, based on which the reliability of the keratoconus matching index as a diagnostic indicator for KC has been demonstrated, achieving an accuracy rate of 97.7%, a sensitivity rate of 91.18%, and a specificity rate of 94.34% [35]. In 2016, based on Corvis ST (CVS), the Corneal Biomechanical Index (CBI) was introduced as a novel measure to differentiate between eyes with keratoconus and those with normal corneas, demonstrating a sensitivity of 94.1% and a specificity of 100% [14]. Similarly, the Chinese version of CBI (cCBI) was recently reported to achieve an AUC of 0.985, a sensitivity of 95.5%, and a specificity of 93.4% [36]. However, in the diagnosis of FFKC, the diagnostic efficacy of individual or combined corneal biomechanical parameters derived from an ORA or Corvis ST is not as robust as in diagnosing keratoconus [37]. Among the explored Corvis ST parameters, SP-A1 exhibited the highest diagnostic efficacy with an AUC of 0.761, a sensitivity of 69.9%, and a specificity of 74.1%, followed by the DA-ratio 2 in Lili’s study [38]. Lei Tian et al. reported that compared with the tomographic parameters, corneal biomechanical parameters are more efficient in the diagnosis of FFKC from normal corneas, and A2DA (corneal deflection amplitude during the second applanation) had the highest diagnosis efficacy with an AUC of 0.766, a sensitivity of 74%, and a specificity of 72%, followed by A1DA (corneal deflection amplitude during the first applanation), A1V (velocity of the corneal apex during the first applanation), and CBI in all tested Corvis ST parameters [39].

In this study, an ROC analysis of all Corvis ST parameters was conducted, revealing Radius (AUC: 0.948) and A2 Time (AUC: 0.938) as the top two parameters for diagnosing FFKC from normal corneas, which deviates from previous findings, probably due to the sample size and population difference. An increase in the sample size and the inclusion of diverse populations are warranted for future validation studies. Although cCBI’s ability to diagnose FFKC from normal corneas is not as robust as its ability to diagnose KC from normal corneas, it outperforms CBI in this context; the results showed that cCBI slightly outperformed CBI in detecting FFKC from the normal population (Table 3), which is believed to benefit from its optimization based on the Chinese population.

It should be noted that the biomechanical parameters discussed so far are based on corneal deformation characteristics of the anterior cornea, incorporating a single or limited number of time points during the entire test, which may not capture the subtle but vital biomechanical abnormalities in FFKC. In fact, most CVS parameters exhibit suboptimal performance in diagnosing FFKC from normal corneas, indicating a deficiency in detecting subtle biomechanical disparities between FFKC and normal corneas through existing parameters. Despite recent advances in combining multiple CVS parameters to enhance KC diagnosis (such as CBI and cCBI), significant biomechanical information may have been lost when relying solely on existing CVS parameters. Furthermore, when air is expelled from the jet orifice by the CVS device, the cornea experiences fluctuating air pressure over time, resulting in various alterations [25]. Previous research consistently indicated that the stress–strain relationship of the cornea following air puff may vary with time due to the visco-elastic nature of the cornea and the temporal changes in air puff pressure, and these variations differ between keratoconus and normal corneas [22,23,24]. Therefore, incorporating deformation characteristics of the cornea in its thickness and the temporal information of such a deformation into artificial intelligence models may enhance the differentiation between FFKC and normal corneas. This hypophysis was supported by the results of this study. By embedding the strain changes of the inner cornea over the entire history of the deformation process, the ML models could successfully distinguish FFKC from the normal corneas with a higher accuracy than existing CVS parameters.

The displacement and strain field data were obtained by an incremental DIC method and previous research has demonstrated that these data could effectively differentiate between keratoconus (KC) and normal corneas [25]. After the DIC analysis of the corneal deformation sequences, biomechanical data from the inner cornea, such as strain changes in the stromal layer, can be detected, while also excluding the influence of whole-eye movement. Therefore, the method was naturally extended to explore the scenario in FFKC where subtle biomechanical changes may be present inside the cornea while obvious biomechanical and morphological changes are still absent at the external surfaces of the cornea.

Given the small sample size and inclusion of time-dimensional data in this study, two single machine learning models and a voting classifier model were selected to distinguish between the two types of corneas. The Random Forest model exhibited the best performance with an AUC of 0.99, consistent with previous studies that highlighted the effectiveness of Random Forest models in differentiating corneas in various states [16,40,41,42,43]. To further enhance outcomes, several studies have advocated for the use of ensemble machine learning models. Ensemble classifiers consistently outperform individual models [44,45]; thus, in this study, an ensemble model was employed to detect FFKC. The voting classifier, comprising a combination of the Naïve Bayes and Random Forest models, outperformed individual models. This outcome can be attributed to the proposed voting ensemble model compensating for the deficiencies of different individual models, thus enhancing the overall performance. Additionally, ensemble models tend to exhibit greater stability compared to single machine learning models, maintaining their performance even when applied to diverse datasets. As is well-known, the accurate identification of FFKC is particularly crucial for determining candidates for refractive surgery. Therefore, the results of this study can provide some reference values for the selection of refractive surgery candidates. However, whether an individual is suitable for refractive surgery is not only related to the presence of FFKC but is also closely related to factors such as corneal thickness, degree of refractive error, and residual corneal thickness post-surgery. Thus, the results of this study can be incorporated as part of the selection system, like the system created by Yoo et al. for refractive surgery candidates [46], aiding in the optimal selection of candidates for refractive surgery.

The integration of the DIC method with machine learning models in this study offers a systematic approach for a more precise analysis of FFKC diagnosis from a novel perspective. The research presents a distinctive methodology, laying the foundation for subsequent incremental improvements. An increased sample size for validation purposes will bolster the robustness of this study. Several limitations in addition to the small sample size need to be noted. First, the cross-sectional design introduces the potential for selection bias. Second, only average displacement and strain values of the corneal cross-section were used in the models, while the patterns of these attributes were not studied due to the limitations of the models adopted. Further investigations should incorporate longitudinal clinical data with multiple follow-up times and explore the pattern changes of the displacement and strain fields using deep learning models. Third, the exclusive use of data from one hospital necessitates further validation across different datasets to ascertain the generalizability of the proposed approach. Rigorous validation studies, including external validation with independent datasets, is crucial to assess the performance, sensitivity, specificity, and clinical utility of the machine learning model in real-world settings. This step should be performed in the future for gaining acceptance and adoption by the medical community. Lastly, clinical implementation and workflow integration of the machine learning models were lacking. User-friendly interfaces and decision-support tools need to be developed in the future to facilitate its adoption and use by ophthalmologists in routine practice.

While there are limitations associated with the small dataset, the potential implications, and applications of the findings in clinical practice are promising and noteworthy. (1) Clinical decision support tool: once validated with larger datasets, the machine learning model has the potential to serve as a valuable clinical decision support tool for ophthalmologists. It can assist clinicians in making more informed diagnoses, leading to early interventions and personalized treatment plans. (2) Enhanced patient care: the accurate diagnosis of forme fruste keratoconus through machine learning can lead to improved patient care by enabling timely interventions, preventing visual impairment, and optimizing treatment outcomes. It can contribute to better patient satisfaction and quality of life. (3) Integration into telemedicine and remote care: machine learning models can be integrated into telemedicine platforms, allowing for remote diagnosis and monitoring of forme fruste keratoconus. This can expand access to specialized care, especially for patients in underserved or remote areas.

## 5. Conclusions

In conclusion, the objective of this study was to develop a framework capable of effectively distinguishing between FFKC corneas and normal corneas, thereby aiding clinicians in enhancing diagnostic efficiency and reducing outpatient visit costs. The proposed method prioritizes accuracy enhancement and reduction of prediction errors in FFKC detection. The experimental findings demonstrated that leveraging displacement and strain data following incremental DIC analysis of Corvis ST images, along with incorporating time domain and inner cornea data, yielded more precise results compared to existing Corvis ST parameters. Moreover, the ensemble classifier, comprising Naïve Bayes and Random Forest models, exhibited superior performance with greater stability. This study delved into the assessment of corneal biomechanical properties through dynamic imaging, emphasizing the versatility of image acquisition across various devices. Subsequently, this diagnostic model can serve as the preliminary groundwork for clinical diagnostic software, guiding the future development of clinical instruments.

## Figures and Tables

**Table 1:** Demographic data of the participants.

Group	Gender (Male/Female)	Age	CCT	bIOP
Normal	35/15	20.08 ± 4.19	558.58 ± 26.55	16.63 ± 2.88
FFKC	39/11	19.74 ± 5.09	530.12 ± 26.51	13.70 ± 2.01
Statistic Value	X^2^ = 0.83	t = 1.12	t = 5.36	t = 5.90
*p* value	0.36	0.26	<0.001	<0.001

**Table 2:** The performance of machine learning models in classifying FFKC and normal corneas in the training and validation datasets.

Dataset	ML Models	Accuracy (%)	Precision	Recall	F1-Score	Sensitivity	Specificity	AUC
Training	Naïve Bayes	86.25	0.82	0.95	0.88	0.95	0.76	0.95
	Random Forest	100.00	1.00	1.00	1.00	1.00	1.00	1.00
	Voting Classifier	86.25	0.82	0.95	0.88	0.95	0.76	0.99
	Logistic Regression	90.00	0.89	0.93	0.91	0.93	0.87	0.97
Validation	Naïve Bayes	85.00	0.73	1.00	0.84	1.00	0.75	0.92
	Random Forest	95.00	0.89	1.00	0.94	1.00	0.92	0.99
	Voting Classifier	85.00	0.73	1.00	0.84	1.00	0.75	1.00
	Logistic Regression	95.00	0.89	1.00	1.00	1.00	0.92	0.94

**Table 3:** The ROC analysis results of top 10 CVS parameters in differentiating FFKC from normal corneas.

Variable	AUC	Sensitivity (%)	Specificity (%)
Radius [mm]	0.948	100.000	87.500
A2 Time [ms]	0.938	75.000	100.000
Max Inverse Radius [mm]	0.932	83.330	100.000
SP A1	0.927	83.330	100.000
cCBI	0.927	91.670	100.000
CBI	0.917	91.670	100.000
SSI2	0.906	91.670	87.500
A1 Time [ms]	0.896	83.330	100.000
SP HC	0.896	91.670	87.500
Integrated Radius [mm]	0.865	75.000	100.000

## Data Availability

The raw data supporting the conclusions of this article will be made available by the authors on request.

## Supplementary Materials

The following supporting information can be downloaded at: https://www.mdpi.com/article/10.3390/bioengineering11050429/s1, Figure S1: The performance of the Naïve Bayes model when incorporating data with different time points; Figure S2: The performance of the Random Forest model when incorporating data with different time points; Figure S3: The feature importance of the machine learning models; Table S1: The 5-fold cross-validation results of the ML models (Final score from 5 folds cross-validation); Table S2: The complete ROC analysis results of Corvis ST parameters in differentiating FFKC from normal cornea.

## Terminology

FFKC	Forme fruste keratoconus, an early form of keratoconus.
KC	Keratoconus, a non-inflammatory eye condition where the cornea progressively thins and bulges into a cone-like shape.
DIC	Digital Image Correlation, an optical method used to measure deformation, strain, and motion on a wide range of materials and structures.
CVS	Corvis ST, a non-contact tonometer equipped with an ultra-high speed Scheimpflug camera used to measure intraocular pressure and assess corneal biomechanics by analyzing the dynamic deformation response of the cornea to an air puff.
AUC	Area under the ROC curve, which plots the true positive rate against the false positive rate.
ROC	Receiver Operating Characteristic, a graphical plot that illustrates the performance of a binary classification model across various threshold settings.
ORA	Ocular Response Analyzer, a device used to measure the biomechanical properties of the cornea, such as corneal hysteresis and corneal resistance factor.
CBI	Corvis Biomechanical Index, a parameter derived from Corvis ST.
cCBI	Corvis Biomechanical Index for Chinese people.
AI	Artificial Intelligence, the simulation of human intelligence in machines programmed to think and learn.
ML	Machine Learning, a subset of AI that enables computers to learn from data and make predictions or decisions without being explicitly programmed.
NB	Naïve Bayes, a probabilistic machine learning algorithm based on Bayes’s theorem with an assumption of independence between features.
RF	Random Forest, an ensemble learning method that combines multiple decision trees to improve predictive accuracy and control overfitting.
TPRK	Trans-Epithelial Photorefractive Keratectomy, a type of laser eye surgery that corrects vision by reshaping the cornea without manually removing the epithelial layer.
LASIK	Laser-Assisted In Situ Keratomileusis, a popular refractive surgery procedure that reshapes the cornea using an excimer laser to correct myopia, hyperopia, and astigmatism.
SMILE	Small Incision Lenticule Extraction, a minimally invasive refractive surgery technique that uses a femtosecond laser to create a lenticule within the cornea, which is then removed through a small incision to correct refractive errors without creating a corneal flap.
