# Supplementary material for: Diagnosis of Forme Fruste Keratoconus Using Corvis ST Sequences with Digital Image Correlation and Machine Learning
Source: Bioengineering (Basel). 2024 Apr 26;11(5):429. doi: 10.3390/bioengineering11050429 (PMC11117575; doi:10.3390/bioengineering11050429)
Supplement: Supplementary file 1 [file bioengineering-11-00429-s001.zip › bioengineering-2929346-supplementary.pdf]

bioengineering

MDPI

Supplementary Materials

Diagnosis of Forme Fruste Keratoconus Using Corvis ST Sequences with Digital Image Correlation and Machine Learning

Lanting Yang [1,2,3,†], Kehan Qi [4,5,†], Peipei Zhang [1,2,3,†], Jiaxuan Cheng [1,2,3], Hera Soha [1,2,3], Yun Jin [6,7], Haochen Ci [6,7], Xianling Zheng [6,7], Bo Wang [6,7], Yue Mei [6,7], Shihao Chen [1,2,3,*] and Junjie Wang [1,2,3,8,*]

[1] National Engineering Research Center of Ophthalmology and Optometry, Eye Hospital, Wenzhou Medical University, Wenzhou 325027, China; landy.yang@foxmail.com (L.Y.); wmu_zhangpeipei@163.com (P.Z.); chengjx0321@163.com (J.C.); h.soha97@hotmail.com (H.S.)
[2] State Key Laboratory of Ophthalmology, Optometry and Visual Science, Eye Hospital, Wenzhou Medical University, Wenzhou 325027, China
[3] National Clinical Research Center for Ocular Diseases, Eye Hospital, Wenzhou Medical University, Wenzhou 325027, China
[4] Shenzhen Institute of Advanced Technology, Chinese Academy of Sciences, Shenzhen 518055, China; qikehan@zju.edu.cn
[5] University of Chinese Academy of Sciences, Beijing 101408, China
[6] State Key Laboratory of Structural Analysis, Optimization and CAE Software for Industrial Equipment, Department of Engineering Mechanics, Dalian University of Technology, Dalian 116023, China; jinyun@mail.dlut.edu.cn (Y.J.); chcc@mail.dlut.edu.cn (H.C.); xianlingzheng@mail.dlut.edu.cn (X.Z.); bo.wang@dlut.edu.cn (B.W.); meiyue@dlut.edu.cn (Y.M.)
[7] International Research Center for Computational Mechanics, Dalian University of Technology, Dalian 116023, China
[8] Department of Ophthalmology, Sichuan Mental Health Center, Mianyang 621054, China
* Correspondence: csh@eye.ac.cn (S.C.); junjie.wang@wmu.edu.cn (J.W.)
† These authors contributed equally to this work.

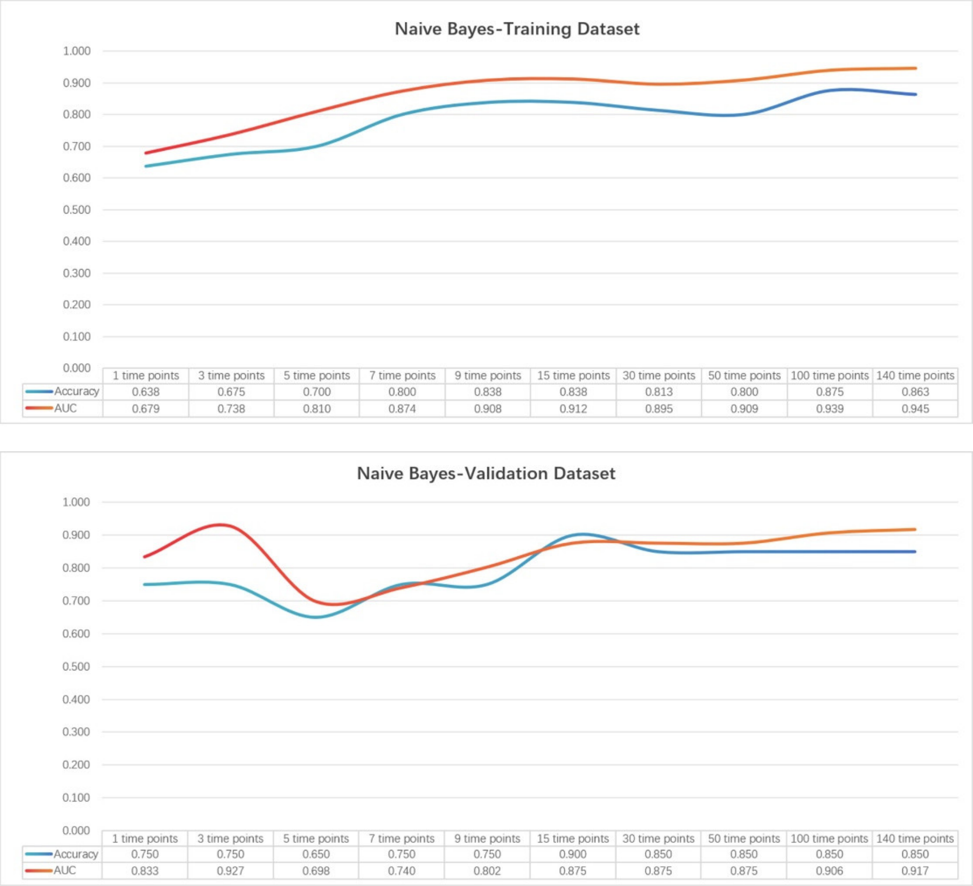

Figure S1. The performance of the Naïve Bayes model when incorporating data with different time points. AUC: area under the receiver operating characteristic curve.

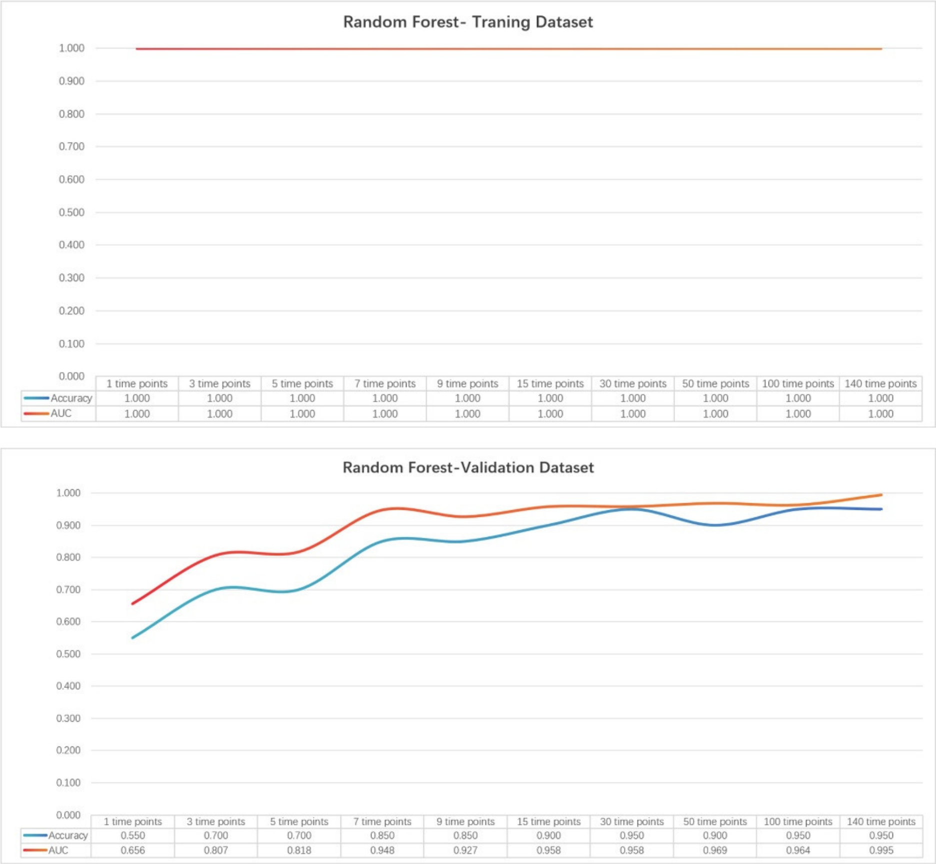

Figure S2. The performance of the Random Forest model when incorporating data with different time points. AUC: area under the receiver operating characteristic curve.

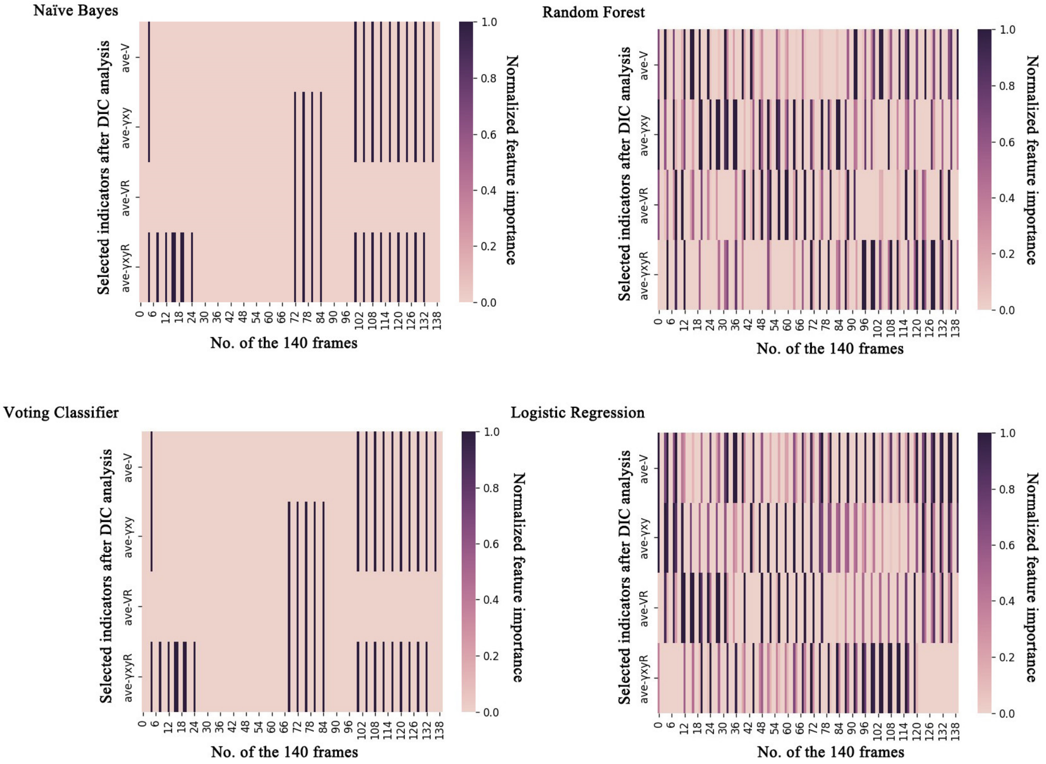

Figure S3. The feature importance of the machine learning models.

Table S1. The 5-fold cross-validation results of the ML models (Final score from 5 folds cross-validation).

ML models	Accuracy (%)	Precision	Recall	F1-Score	Sensitivity	Specificity	AUC
Naïve Bayes	78.00	0.71	0.91	0.79	0.91	0.63	0.82
Random Forest	83.00	0.85	0.83	0.82	0.83	0.89	0.90
Voting Classifier	78.00	0.71	0.91	0.79	0.91	0.63	0.90
Logistic Regression	75.00	0.72	0.88	0.78	0.88	0.64	0.83

Table S2. The complete ROC analysis results of Corvis ST parameters in differentiating FFKC from normal cornea.

Variable	AUC	Sensitivity (%)	Specificity (%)
Radius [mm]	0.948	100.000	87.500
A2 Time [ms]	0.938	75.000	100.000
Max Inverse Radius [mm]	0.932	83.330	100.000
SPA1 [mmHg/mm]	0.927	83.330	100.000
cCBI	0.927	91.670	100.000
CBI	0.917	91.670	100.000
SSI2	0.906	91.670	87.500
A1 Time [ms]	0.896	83.330	100.000
SPHC [mmHg/mm]	0.896	91.670	87.500
Integrated Radius [mm]	0.865	75.000	100.000
AP1 [mmHg]	0.854	66.670	100.000
Whole Eye Movement Max [ms]	0.854	91.670	75.000
PachySlope [μm]	0.844	66.670	100.000
A2 dArc Length [mm]	0.786	75.000	87.500
DA Ratio Max 2mm	0.771	83.330	75.000
DA Ratio Max 1mm	0.771	83.330	75.000
AP2 [mmHg]	0.760	66.670	100.000
A1 Deflection Length [mm]	0.760	50.000	100.000
dArc Length Max [mm]	0.750	75.000	75.000
A2 Deflection Area [mm^2]	0.729	50.000	100.000
bIOP [mmHg]	0.724	50.000	100.000
HC dArc Length [mm]	0.724	100.000	37.500
A1 Deflection Amp [mm]	0.719	83.330	62.500
HC Time [ms]	0.708	58.330	100.000
Def. Amp. Max [mm]	0.708	58.330	87.500
HC Deformation Amp. [mm]	0.708	58.330	87.500
HC Deflection Amp. [mm]	0.698	58.330	75.000
Deflection Amp. Max [mm]	0.693	75.000	62.500
simK3mm [mm]	0.688	58.330	87.500
A2 Deflection Amp. [mm]	0.677	50.000	100.000
A2 Deflection Length [mm]	0.677	83.330	62.500
Deflection Amp. Max [ms]	0.677	100.000	50.000
ZonalK7mm [mm]	0.677	66.670	87.500
HC Deflection Area [mm^2]	0.635	58.330	75.000
A1 dArc Length [mm]	0.630	91.670	37.500
A1 Deflection Velocity [m/s]	0.620	75.000	50.000
A2 Deflection Velocity [m/s]	0.615	41.670	100.000

A1 Deformation Amp. [mm]	0.615	83.330	50.000
A1 Velocity [m/s]	0.609	33.330	100.000
A2 Deformation Amp. [mm]	0.583	50.000	87.500
Whole Eye Movement Max [mm]	0.583	91.670	37.500
A2 Velocity [m/s]	0.573	41.670	100.000
SSI	0.563	50.000	75.000
Peak Dist. [mm]	0.547	58.330	62.500
ARTh	0.542	41.670	100.000
HC Deflection Length [mm]	0.521	50.000	75.000
A1 Deflection Area [mm^2]	0.510	75.000	0.000
